# Differences in body dissatisfaction between individuals with and without stigma toward obesity: A study of preadolescents and adolescents

**DOI:** 10.1007/s40519-024-01693-1

**Published:** 2024-10-08

**Authors:** Adriana Amaya-Hernández, Mayaro Ortega-Luyando, Juan Manuel Mancilla-Diaz, Georgina Alvarez-Rayón, Michelle Cruz-Navarro, Alejandro Pérez-Ortiz

**Affiliations:** 1 Nutrition Research Group, Facultad de Estudios Superiores Iztacala, División de Investigación y Posgrado, Universidad Nacional Autónoma de México, Av. de los Barrios #1, Los Reyes Iztacala, 54090 Tlalnepantla, Mexico Mexico; 2 Mexico City, Mexico

**Keywords:** Obesity, Children, Adolescents, Body image, Stigma

## Abstract

**Introduction:**

Previous studies have focused on understanding the biopsychosocial implications of obesity stigma and have made proposals to minimize its negative consequences, as well as recommendations to eliminate or reduce this stigma; however, knowing which individuals stigmatize obesity and why will allow us to have a broader picture of stigmatization and thus help in planning interventions with greater impact.

**Objective:**

The aims were to describe the stigmatization toward obesity in preadolescents and adolescents and to determine whether there are differences in body dissatisfaction, abnormal eating behaviors and self-esteem among those with and without stigma toward obesity.

**Methods:**

A total of 307 preadolescents and 349 adolescents answered a set of questionnaires that evaluated abnormal eating behaviors, body dissatisfaction, self-esteem and stigma.

**Results:**

Fifty-nine percent of the participants stigmatized individuals with obesity, with preadolescents having the greatest stigma levels. Differences were observed only in body dissatisfaction, where the group of preadolescents who stigmatized individuals with obesity and the group of adolescents who did not stigmatize individuals with obesity reported higher levels of body dissatisfaction.

**Conclusion:**

Obesity is stigmatized at early ages, regardless of sex; however, preadolescents with stigma toward obesity and adolescents without stigma toward obesity have greater body dissatisfaction, indicating that body dissatisfaction plays a crucial role in the stigmatization of obesity.

**Level of evidence:**

Level V, cross-sectional analytical study.

## Introduction

Stigma toward obesity can be defined as discriminatory acts and ideologies targeted toward individuals because of their weight and size [1]. People with obesity experience stigma, mainly by family members, by health professionals and in scholarly environments; similarly, stigma has multiple important implications for physical and psychosocial health [2].

This evidence has yielded recommendations aimed at eliminating or reducing stigma toward obesity, such as legal regulations in public policies and prevention programs. Although these initiatives may decrease stigma behaviors and attitudes, it is necessary to consider that there are multiple factors that favor or trigger stigma [3]. Therefore, in addition to promoting anti-stigma attitudes, it is necessary to study those with stigma toward obesity; however, we are not aware of previous studies that have analyzed people who stigmatize obesity or that have distinguished between those who stigmatize and those who do not. These types of studies would make it possible to understand all the elements related to stigma to prevent its development.

Although there are no conclusive results concerning which developmental stage is more vulnerable to the development of stigma attitudes and behaviors, it has been reported that childhood and adolescence are critical stages [2]. On the other hand, stigma is the result of the internalization of body ideals that reject obesity and encourage thinness; therefore, people with stigma toward obesity may engage in disordered eating behaviors that could affect their self-esteem, as the literature has shown [2]. Therefore, the objectives of this study were as follows: first, to describe the stigma toward obesity in preadolescents and adolescents; and second, to determine whether there are differences in body image satisfaction, abnormal eating behaviors and self-esteem among individuals who stigmatize individuals with obesity and those who do not. The hypotheses of this study were as follows: (a) preadolescents and adolescents will have stigma toward obesity and (b) those with stigma toward obesity will report greater body dissatisfaction, abnormal eating behaviors, and low self-esteem.

## Materials and methods

### Participants

The sample was non-probabilistic and intentional, comprising 656 students: 307 were preadolescents (*n* = 157 girls, *n* = 150 boys), and 349 were adolescents (*n* = 196 girls, *n* = 153 boys), all of whom were inhabitants of the metropolitan area of Mexico City and from families with lower-middle income levels. The inclusion criteria for students were as follows: (a) were between 9 and 15 years of age, and (b) gave consent to participate in the study. The exclusion criteria for students were as follows: (a) did not attend school during data collection and (b) did not have anthropometric measurements or did not answer more than 10% of the questions.

### Variables and measures

#### Abnormal eating behaviors

The Children's Eating Attitudes Test assesses the presence of symptoms and characteristics of eating disorders and has 26 items with six-point Likert-type response options. This instrument was adapted to the Mexican population by Escoto and Camacho [4], who reported that the test had adequate internal consistency (*α* = 0.82). An *alpha* value of 0.77 was obtained in the present study.

#### Body dissatisfaction

The Body Shape Questionnaire-16 assesses satisfaction with body image and is composed of 16 items with six-point Likert-type response options. This instrument was adapted to the Mexican population by Amaya [5], who reported that the questionnaire had an internal consistency of 0.90; however, in the present study, an *alpha* of 0.92 was obtained.

#### Self-esteem

The Self-Esteem Test for Children and Adolescents adapted for the Mexican population by Caso [6] comprises 21 items with five-point Likert-type response options. In the study of a Mexican population, Caso reported a reliability index of 0.82, whereas in the present study, an *alpha* of 0.88 was obtained.

#### Stigma

Six drawings by Richardson et al. [7] illustrating a "physically healthy" child/adolescent, four children/adolescents with some type of disability—the use of crutches, use of a wheelchair, presence of a facial scar or the presence of one arm—and a child/adolescent with obesity were used. All individuals in the drawings had the same dimensions, facial expressions, and clothing; notably, the drawings were adapted to Mexican physical features, and two versions of these drawings were used, one with male/female preadolescents and the other with male/female adolescents. The purpose of these drawings was for the participants to observe the physical characteristics of the individuals in the drawings in detail and order them according to their preferences on the basis of the following questions: *"Which of them would you choose as your first best friend?*”; once they made a selection, the participants were asked *“Of those who are left over, who would you choose as your second best friend?”* and so on until each drawing was assigned a number from 1 to 6. For analysis purposes, participants who ranked the individual with obesity in 4th, 5th or 6th place were assigned to the group with stigma toward obesity, whereas those who ranked the individual with obesity in 1st, 2nd or 3rd place were assigned to the group that without stigma toward obesity.

#### Body mass index (BMI)

Anthropometric measurements (weight and height) were carried out via standardized equipment [scale and stadiometer, recommended by the International Society for the Advancement of Kinanthropometry]. The participants were subsequently classified as underweight, normal weight, overweight or obese according to the World Health Organization reference curves.

### Procedure

This was a cross-sectional, non-experimental research; the research protocol was submitted for review by the ethics committee of the Facultad de Estudios Superiores Iztacala of the National Autonomous University of Mexico and was approved under the registration number CE/FESI/062018/1256. The authorities of the educational institutions were subsequently asked to participate, and the research protocol was explained. Once permission from the school principals was obtained, data collection was conducted between March and April 2022.

Informed consent and a personal data sheet were sent to the parents, and assent was obtained from the participants, who completed the four questionnaires and evaluated the two versions (male and female) of the Richardson drawings. The questionnaires were completed in the classroom, and the session lasted approximately 1 h. Finally, a trained health professional measured the height and weight of each participant.

At the end of the data collection, a report was delivered to the authorities of each school, and all the participants, parents and teachers received psychoeducation concerning body diversity and assertive ways to cope with body discrimination.

### Statistical analysis

All the data were analyzed with the statistical package SPSS (version 20). A descriptive analysis of the sample was performed, as were a Chi-square test of independence and Student's t test, to determine differences between the groups (stigma *vs*. no stigma). Statistical significance was determined with a *p* value equal to or less than 0.05.

## Results

In both age groups, approximately 40% of the participants were overweight or obese, whereas 59.9% of the sample had stigma toward obesity, meaning that 393 participants (207 [84.5%] preadolescents; 186 [68.1%] adolescents) ranked the boy/girl with obesity in last place (see Table 1). Notably, 4 out of 10 participants with stigma toward obesity met the BMI classification of overweight or obesity. Additionally, significant differences between the preadolescent and adolescent groups were found in obesity stigma [*X*^2^(1) = 18.87; *p* = 0.001]; therefore, subsequent analyses were performed by age group. Differences between the sexes were analyzed in those with and without stigma toward obesity, and no statistically significant differences were found between the groups of preadolescents [*X*^2^(2) = 0.95; *p* = 0.620] and adolescents [*X*^2^(2) = 4.24; *p* = 0.120].

**Table 1:** Demographic, body weight classification and stigma features

	Preadolescents	Adolescents
	*n* = 307	*n* = 349
Sex		
Female	157	196
Male	150	153
Age		
Median	10.33	12.99
SD	0.79	0.90
Range	9–11	12–15
BMI		
Underweight	3 (1.0%)	3 (0.9%)
Normal weight	176 (57.3%)	212 (60.7%)
Overweight	75 (24.4%)	85 (24.4%)
Obesity	53 (17.3%)	49 (14.0%)
Stigma		
Yes	207	186
No	38	87

SD: standard deviation; BMI: body mass index

In both the preadolescent and adolescent groups, the drawings of the child in a wheelchair and the child with a normal weight were the first two to be chosen, with the latter being the most preferred. In the group of preadolescents, the child with obesity was the last to be selected, and in the group of adolescents, the drawings of with obesity and one-armed children were the last to be selected.

With respect to the differences between the participants who did and did not have stigma toward obesity, we observed significant differences in body mass index in the preadolescent group and in body dissatisfaction in the preadolescent and adolescent groups; however, preadolescents with stigma toward obesity had greater body dissatisfaction than did those without stigma toward obesity, and in the adolescent group, the participants without stigma toward obesity had greater body dissatisfaction than those with stigma toward obesity (see Table 2).

**Table 2:** Comparisons of abnormal eating behaviors, body dissatisfaction and self-esteem between the groups of preadolescents and adolescents with and without stigma toward obesity

	Stigma	Nonstigma		
	*Preadolescents*			
	*n* = 207	*n* = 38	*t*	*p*
Body mass index	19.47 (3.76)	18.07 (3.22)	2.38	0.02
Abnormal eating behaviors	14.32 (9.50)	12.05 (7.29)	− 1.40	0.16
Body dissatisfaction	33.13 (13.08)	28.24 (12.94)	− 2.14	0.03
Self-esteem	53.76 (6.65)	54.02 (6.94)	0.212	0.83

	*Adolescents*			
	*n* = 186	*n* = 87	*t*	*p*
Body mass index	21.12 (3.93)	22.04 (4.33)	− 1.67	0.08
Abnormal eating behaviors	9.53 (6.68)	10.89 (9.31)	1.22	0.22
Body dissatisfaction	29.21 (13.81)	34.72 (20.26)	2.62	0.00
Self-esteem	77.05 (17.97)	80.77 (15.29)	1.66	0.09

## Discussion

A total of 40% of the sample was overweight or obese, which is consistent with international prevalence data that emphasize that obesity is a serious health problem [3]. However, BMI has been reported to be an inaccurate measure for determining health risks related to “overweight” or “obesity” since it does not consider muscle mass, bone density, or genetics, among other factors [8]. This is why it is important to deconstruct false associations between high BMI and low-quality health, since this favors stereotypes, prejudice and body stigma.

On the other hand, 59.9% of the preadolescents and adolescents (mostly those with a normal weight) who participated in this study had stigma toward obesity; this finding is consistent with the findings of previous studies [2], showing evidence that normalization of stigma toward obesity emerges in the early developmental stages. Furthermore, both preadolescents and adolescents had stigma toward obesity, and these findings support the first hypothesis of this study. A possible explanation for these results is that beginning at early ages (preadolescence), humans are taught the socially acceptable weight and physical appearance associated with thinness [2] and consequently develop early attitudes of rejection toward body shapes and weights that do not fit with those social standards.

The results obtained in this research are in line with those of previous studies [9] that mention that, regardless of the stage of development or sex, illustrations of individuals with normal weights and in wheelchairs are generally ranked first, whereas individuals with obesity are ranked last. A possible explanation for these results is suggested by Bos et al. [1], who reported that stigma arises from several cognitive representations that trigger emotional and behavioral reactions. Among these representations are the “Perceived severity” and “Onset controllability” representations. Perceived severity is commonly observed in individuals with severe disabilities, such as those requiring wheelchair use, which can yield emotional ambivalence, resulting in a mix of anxiety and sympathy, with sympathy being the emotion that stands out in social interactions. This theoretical proposal may explain why, in this study, participants in both age groups ranked the individual in the wheelchair in first place.

On the other hand, onset controllability refers to the belief that the stigmatized condition is largely the responsibility of the person (e.g., smokers who develop lung cancer); therefore, the presence of stigmatizing behavior is the result of the assumption that a person could have controlled his or her condition but did not want to [1], which could explain the ranking of individual with obesity in last place.

In response to the second objective, it was observed that preadolescents with stigma toward obesity had higher scores for body dissatisfaction than did those without stigma toward obesity. Additionally, adolescents without stigma toward obesity had higher scores for body dissatisfaction than did those with stigma toward obesity. With these results, we partially reject the second hypothesis. To explain these results, it is necessary to consider body dissatisfaction and its relationship with self-stigma.

Body dissatisfaction arises from negative thoughts and feelings about one’s body, since it is believed that socially imposed standards on body esthetics are not met, which means that one does not have a thin or slender body [10]. On the other hand, Pryor and Reeder [1] reported that self-stigma is characterized by the idea of being exposed to stigma because one possesses or has the idea of having traits of the stigmatized condition. Two types of negative reactions resulting from self-stigma are explained through the dual-process model of reactions to perceived stigma: implicit and explicit reactions. The former are characterized by immediate aversion and automatic negative reactions, whereas the latter are characterized by controlled and reflective reactions, allowing negative reactions to be attenuated or dissipated.

Given the above, it could be inferred that preadolescents with stigma toward obesity and who had greater body dissatisfaction had an implicit reaction, whereas adolescents without stigma toward obesity and who had greater body dissatisfaction had an explicit reaction.

## Strengths and limitations

In view of the limitations of this study, personal interviews should be performed to ensure that participants who ranked the obesity illustration in last place did so due to stigma. Additionally, other variables that may trigger obesity stigma, such as family, media, peers, or coping skills [2, 3], should be evaluated, and longitudinal designs and statistical methods that take into account potential confounding variables should be used to ensure the generalizability and validity of the results.

We are not aware of previous studies that have compared groups with and with stigma toward obesity; this result could indicate that appearance and attractiveness are especially important during early developmental stages, such as preadolescence, regardless of sex. In addition, body dissatisfaction plays a relevant role in the stigmatization of obesity.

In this study, a first approach was taken to understand who stigmatizes obesity and what psychological variables are more common in these individuals, concluding that the presence of body dissatisfaction is associated with the stigmatization of obesity by preadolescents but not by adolescents.

## What is already known about this subject?

- More than half of people with obesity experience stigmatization, mainly by family, by health professionals or in school environments.
- The consequences of weight stigma affect body satisfaction, self-esteem and eating behavior.
- Public health initiatives exist to counteract the emotional and health consequences of weight stigma.

## What does this study add?

- This study paid special attention to preadolescents and adolescents, who negatively view people with obesity and participate in stigmatization.
- Body dissatisfaction prevents stigmatization by adolescents but not by preadolescents.
- Preadolescents have greater stigma than adolescents do.

## Data Availability

The datasets generated during and/or analyzed during the current study are available from the corresponding author on reasonable request.
